# Present and potential future critical source areas of nonpoint source pollution: a case of the Nakdong River watershed, South Korea

**DOI:** 10.1007/s11356-021-12976-w

**Published:** 2021-04-20

**Authors:** Mijin Seo, Joonghyeok Heo, Yongseok Kim

**Affiliations:** 1 Nakdong River Environment Research Center, National Institute of Environmental Research, 24-11, Gukgasandan-daero 52, Daegu, 43008 South Korea; 2 University of Texas-Permian Basin, 4901 E. University Blvd., Odessa, TX 79762 USA

**Keywords:** Nakdong River watershed, NPS pollution, CSAs, Past rainfall, Climate change, RCP

## Abstract

Identifying critical source areas (CSAs) is the first step to effectively managing nonpoint source (NPS) pollution. Increasing variability in climate can affect identification of CSAs. In this study, we identified present and future CSAs of NPS pollution in the Nakdong River watershed and examined how climate change will influence the identification of CSAs. Nine NPS pollution-related factors affecting the watershed environment and water quality were considered. These factors were rescaled through a min-max normalization to propose an index system that ranks basins based on the sensitivity of basins to climate change on identifying CSAs. For analyses, past rainfall was replaced with future rainfall under two RCP scenarios, RCP 2.6 and RCP 8.5. Results showed insignificant differences in the spatial distribution of CSAs between the present and the future and between the future scenarios. Basins that are on or adjacent to the Nakdong River mainstream were mainly identified as CSAs, in addition to many basins of the Geumho and Nam rivers. Highly ranked CSAs including the level 1 CSAs, were mainly distributed in the mid- and downstream areas of the Nakdong River, indicating high need of NPS pollution management. This study can provide a foundation for the effective management of NPS pollution in the present and the future.

## Introduction

Nonpoint source (NPS) pollution by diffuse sources of pollutants on the landscape is difficult to manage due to the nature of diffuse sources presenting large spatial and temporal variabilities. The management of NPS pollution has however become essential in South Korea due to growing proportion of NPS pollution over the total water pollution (Kang et al. 2014). Managing land areas to control NPS pollution requires substantial efforts and large costs (Kang et al. 2013). To effectively manage NPS pollution, critical source areas (CSAs) susceptible to NPS pollution should be first to be identified and systematically managed. The identification of CSAs of NPS pollution has been studied using various methods: laboratory experiments (Doppler et al. 2014; Lucci et al. 2012), water quality change analysis (Kang et al. 2013), correlation analysis (Kal et al. 2019), overlay analysis using the geographic information system (GIS) (Orlikowski et al. 2011; Park et al. 2010), and analytic hierarchy process (AHP) involving expert surveys (Jang et al. 2012; Shin et al. 2007). Various models have been also used to identify areas in need of NPS pollution management (Dong et al. 2018; Ghebremichael et al. 2010; Liu et al. 2016; Niraula et al. 2012, 2013; Shang et al. 2012; Winchell et al. 2015; Yi et al. 2015; Zhuang et al. 2016). However, laboratory experiments are often limited in research scope and water quality monitoring studies are time-consuming and require a large workforce and costs (Lee et al. 2012; Park et al. 2015). The AHP method has uncertainties associated with variable independence and hierarchy (Jang et al. 2012; Shin et al. 2007). Modeling approaches are often challenged by the difficulties in the composition of input data and long processing time (Jang et al. 2012; Munafo et al. 2005). Identifying CSAs using such complex and limited methods can delay the progress of NPS pollution management. A simple and parsimonious approach using NPS pollution-related factors can be an effective method for NPS pollution management.

The Ministry of Environment (MOE) of South Korea provides governmental authority in managing CSAs of NPS pollution, which are defined as areas with (1) >50% NPS pollution among watersheds not meeting water quality standards, (2) a population above one million, (3) industrial complexes, (4) NPS pollutants harming the environment, or (5) the necessity of geographical management (Article 54 of the Water Environment Conservation Act and Article 76 of the Enforcement Degree of the Water Environment Conservation Act). The Nakdong River watershed, the second largest watershed in South Korea, has the basins including Andong, Yangsan, and Kimhae cities which are identified as CSAs under the Articles 54 and 76. Other basins of the Nakdong River watershed have been proposed to be designated as CSAs through several studies performed by the MOE (Kim et al. 2012; MOE 2016a; Park et al. 2013, 2015). The Korea Environment Corporation (KECO) (2018) has also studied CSAs that are in need for the management of agricultural NPS pollution in the Nakdong River watershed. However, most of these studies targeted at large scale (whole peninsula of South Korea) or at micro scale (only several parts of a watershed) as the study area. The prioritization of CSAs can change depending on the spatial scope of the analysis. At large scale, high priorities of CSAs can be given to a certain watershed with many pollution factors such as a high ratio of impervious areas and high population density and potential CSAs in other watersheds can be excluded. At micro scale, it is difficult to figure out whether the selected study area has a higher priority than other areas. Because each watershed has different characteristics, selecting a target watershed with similar characteristics can lead to an appropriate identification of CSAs of NPS pollution in the watershed.

Studies of CSAs of NPS pollution often relied on past rainfall as a forcing term. However, rainfall variation due to climate change can lead to changes in the discharge characteristics of NPS pollutants (Seo et al. 2019a), which can affect changes in CSAs of NPS pollution. The Intergovernmental Panel on Climate Change established four Representative Concentration Pathways (RCP) climate change scenarios (8.5/6.0/4.5/2.6), from high-concentration greenhouse gas emission scenario to mitigation scenario. Under the RCP system, the Korea Meteorological Administration (KMA) provides the global (135 km resolution), Korean Peninsula (12.5 km resolution), and South Korea (1 km resolution) future climate data until 2100. The National Institute of Meteorological Research (NIMR) (2012) predicted the increases of temperature and rainfall and the aggravation of climate change for the Korean Peninsula. Accordingly, studies have demonstrated the negative impact of climate change on NPS pollution in South Korea (Ahn et al. 2014; Han et al. 2017; Jang and Kim 2017). In this regard, identifying CSAs under climate change and preparing for changes in CSAs are necessary steps to an effective management of NPS pollution. However, the impact of climate change was only considered in few studies of CSAs and most of them were modeling approaches (Liu et al. 2017; Renkenberger et al. 2016; Shrestha et al. 2019).

This study attempted to identify present and future CSAs and to examine spatial variability of CSAs under future climate scenarios in the Nakdong River watershed, South Korea. The CSAs where management should be prioritized were identified using a parsimonious method which utilizes NPS pollution-related factors to rank CSAs and to analyze spatial distributions of CSAs. The novel method can help policy makers effectively manage CSAs for NPS control against present and future climates.

## Materials and methods

### Study area

**Fig. 1:**
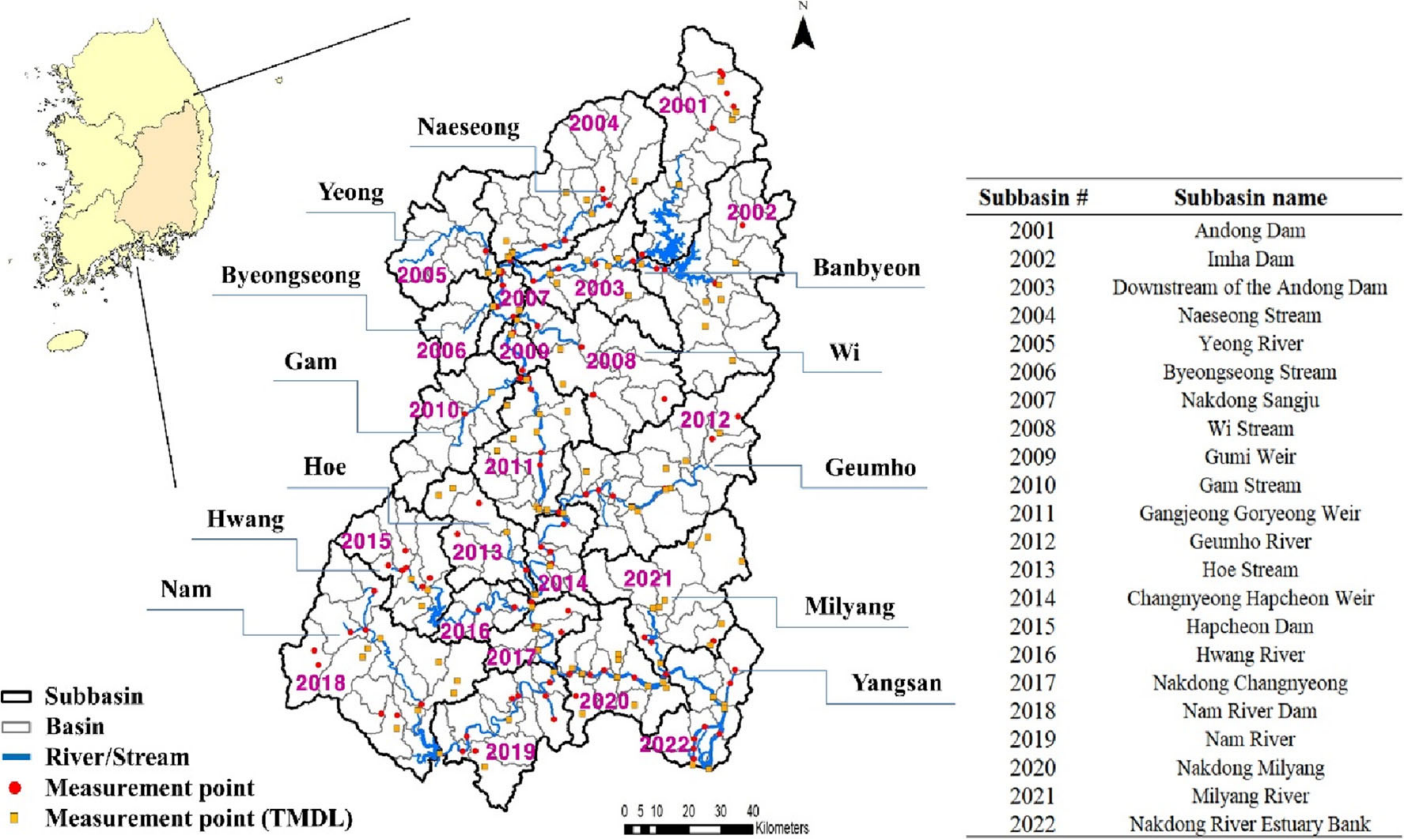
Nakdong River watershed consisting of 22 subbasins and 195 basins. The first letter of each word of subbasin and basin names was capitalized in the study

The watershed of the Nakdong River, the longest river in southeastern South Korea, covers an area of 23,384 km^2^. The Nakdong River watershed consists of 22 subbasins which are further split to 195 basins where a basin is the smallest unit (Fig. 1). Major tributaries (e.g., Banbyeon, Naeseong, Geumho, Nam, and Milyang) flow into the Nakdong River and water quality monitoring stations are operated in the tributaries and mainstream. The climate of the Nakdong River watershed is hot and humid in the summer and cold and dry in the winter. The annual rainfall is on average 1200 mm and approximately 60% of the total rainfall occurs during rainy season from June to September due to large seasonal variation.

### Data collection and build-up

Various NPS pollution-related factors affecting the watershed and water quality environments were considered in the analysis. These factors were selected by referring to related studies (Kim et al. 2012, 2019; Lee et al. 2012; NRERC 2018; Orlikowski et al. 2011; Park et al. 2010). Nine factors were selected, that is, load factors (1) NPS pollution load per unit area, (2) residential/industrial NPS pollution load per unit area; water quality factors (3) mean water quality, (4) excess percentage of water quality standards, (5) water quality index; and watershed environment factors (6) impervious area, (7) soil slope, (8) topsoil type, (9) rainfall.

The loads (factors 1 and 2) were determined for each basin using estimated load data of biological oxygen demand (BOD), total nitrogen (TN), and total phosphorus (TP), based on the technical guidelines for Korean total maximum daily loads (TMDLs) (NIER 2019). The mean water quality (factor 3) and the excess percentage of water quality standards (factor 4) were determined for each basin using water quality data including BOD, chemical oxygen demand (COD), TP, suspended solid (SS), and total organic carbon (TOC) at 253 monitoring stations in the Nakdong River watershed. The variables were chosen depending on the availability of data and the existence of water quality standards. The data were considered for rainy season (June to September) which can reflect the impact of NPS pollution and were obtained from the Water Environment Information System (http://water.nier.go.kr). The excess percentage of water quality standards for each basin was calculated from the difference between the mean water quality and water quality standards:

1 $$ \mathrm{Excess}\ \mathrm{percentage}\ \left(\%\right)=\frac{\left({V}_i\hbox{--} {V}_s\right)}{V_s}\times 100 $$

where V_i_ is the mean water quality of the basin i, and V_s_ is the water quality standards of the basin i. The water quality index (WQI; factor 5) is a water quality score that is used to classify various and complex water quality states. It is divided into five levels: satisfactory (80–100), adequate (60–79), normal (40–59), cautious (20–39), and poor (0–19). The WQI was calculated for each basin as follows:

2 $$ \mathrm{WQI}=100-\sqrt{\frac{\left({F}_1^2+{F}_2^2+{F}_3^2\right)}{3}} $$

where F_1_, F_2_, and F_3_ are obtained from the number, number of times, and degree of water quality variables not meeting standard values, respectively. More details can be found in the Real Time Water Quality Information System (http://www.koreawqi.go.kr). For water quality factors, to ensure that basins without water quality monitoring stations were not excluded from the identification of CSAs, the mean values of their subbasins were applied to each basin (Kim et al. 2012).

The impervious area (factor 6) was determined using land cover data from the Environmental Geographic Information Service. The total area, including the urban areas and greenhouses in agricultural areas, was calculated for each basin. The soil slope (factor 7) and the topsoil type (factor 8) were determined using soil data from the Rural Development Administration. A steep slope can negatively affect water quality because more pollutants are discharged into the river due to soil losses. Thus, except for the slope of 60–100% including mountainous areas, the total area in the second steepest slope ranges (30–60%) was calculated for each basin. Regarding the topsoil type, clay loam soils belonging to type D of the Natural Resources Conservation Service hydrologic soil groups (HSGs) exhibit the lowest infiltration and highest surface runoff among the four HSGs (MOE 2016b). The total area of clay loam and silty clay loam soils was estimated for each basin. The rainfall (factor 9) was determined using past and future rainfall data obtained from 24 weather stations of KMA in the Nakdong River watershed. Average annual rainfall was calculated for each weather station and the rainfall of each basin was then calculated via weighted mean using the Thiessen polygon method. To assess the variability of CSAs of NPS pollution due to future climate change, RCP 2.6, the best scenario in which the Earth can recover from the impact of human activities on its own, and RCP 8.5, the worst scenario in which greenhouse gas emissions continue to occur, were considered as future scenarios. The Korean Peninsula (12.5 km resolution) rainfall data from 2021 to 2100 were processed for both scenarios. The nine factors mentioned above were determined using data available from 2008 to 2018 and most recently updated data.

### Analysis method

The impact of NPS pollution on the 195 basins of the Nakdong River watershed was analyzed by normalizing each factor. Normalization is a method that can be used to evaluate factors on the same basis by scaling each factor to have values between 0 and 1. The values of each factor were normalized with the min-max method and expressed as percentages (I) in Eq. (3). A higher percentage indicates a higher impact of NPS pollution. The final index (FI) was calculated for each basin based on the sum of factors multiplied by an equal weight as Eq. (4). The CSAs were identified by ranking the FI values of basins.

3 $$ \mathrm{I}=\frac{\left({V}_i-{V}_{min}\right)}{\left({V}_{max}-{V}_{min}\right)}\times 100 $$

4 $$ \mathrm{FI}=\mathrm{factor}1\times \mathrm{w}1+\mathrm{factor}2\times \mathrm{w}2+\cdots +\mathrm{factor}9\times \mathrm{w}9 $$

where V_i_ is the value of the basin i, V_min_ and V_max_ are the minimum and maximum values among all 195 basins, respectively, and w is the weight of each factor. The method using NPS pollution-related factors to identify CSAs can be easily applied to large watersheds and was utilized in several studies (Jang et al. 2012; Kim et al. 2012, 2019; Lee et al. 2012). Among the 195 basins, the top 30% (ranks 1–59) in high need of NPS pollution management were identified. The identified CSAs were further divided into three subgroups to analyze the rankings: level 1 (1%–10%; ranks 1–20), level 2 (11%–20%; ranks 21–39), and level 3 (21%–30%; ranks 40–59). Results were obtained for BOD and TP and for total nonpoint (TNP), which means the average of considered water quality variables. The present and future CSAs were determined by applying past and future rainfall data, respectively, under the same factor conditions. For the present results, the reliability of the identified CSAs was evaluated in comparison with CSAs in the Nakdong River watershed that were reported by the MOE and KECO, as mentioned in the “Introduction” section.

### Load duration curve analysis

Load duration curve (LDC) analysis can be used to identify areas that require NPS pollution management based on the comparison between the target and measured loads estimated using target and measured water quality and flow data. Related studies have shown that LDC analysis is suitable for the identification of CSAs of NPS pollution (Jang et al. 2018; Kim 2014; Park et al. 2015, 2017). Park et al. (2015) used LDC analysis to compensate for temporal variations of flow and water quality in the identification of CSAs. This study also used LDC analysis to assess the reliability of the identified CSAs.

The LDC was drawn by exclusively using Korean TMDL monitoring stations that measure both water quality and flow. The MOE has been operating a total of 102 monitoring stations in the Nakdong River watershed (Fig. 1); the target water quality regarding BOD and TP has been set and systematically managed under the Korean TMDL program. The LDC was drawn using BOD, TP, and flow data collected at each of the 102 monitoring stations from 2008 to 2018. The flow intervals (*x*-axis) of the LDC are divided into high flows (0–10%), moist conditions (10–40%), mid-range flows (40–60%), dry conditions (60–90%), and low flows (90–100%), where each interval indicates different pollutant properties. The United States Environmental Protection Agency US EPA (2007) reported that the high flow and moist condition intervals generally reflect the impact of NPS pollution. Jung et al. (2011) also mentioned that the deterioration of the water quality in the intervals above moist conditions could be explained with NPS pollution. In LDC analysis, the water quality is deemed unsatisfying if the measured load exceeds the target load by more than 50% for each interval. In this study, the LDC was analyzed for the excess rates in the high flow and moist condition intervals. Basins including unsatisfying monitoring stations were identified and the top 10% (ranks 1–10) basins in high need of NPS pollution management were identified by ranking normalized excess rates. The basins were analyzed in comparison with the present CSAs.

## Results and discussion

### Factor analysis

**Fig. 2:**
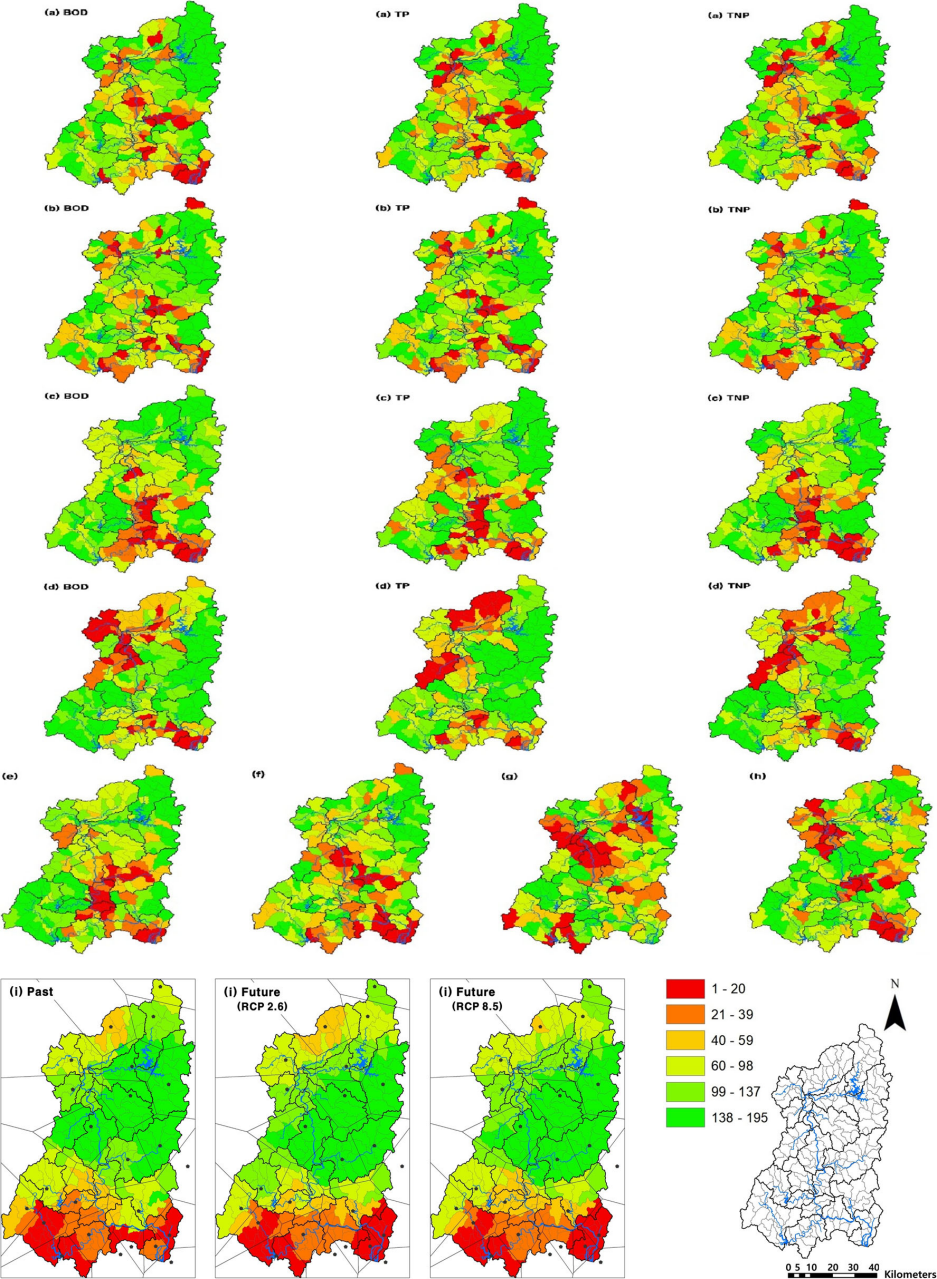
Influence of each factor on NPS pollution in the Nakdong River watershed. **a** NPS pollution load per unit area, **b** residential/industrial NPS pollution load per unit area, **c** mean water quality, **d** excess percentage of water quality standards, **e** water quality index, **f** impervious area, **g** soil slope (30–60%), **h** topsoil type (clay loam), and **i** rainfall. Left, middle, and right figures from (**a**) to (**d**) mean BOD, TP, and TNP, respectively

The impact of each factor on NPS pollution in the Nakdong River watershed was examined by ranking normalized values (Fig. 2). The NPS pollution load accounted for 70–80% of the total load. The land and livestock pollution load accounted for 98–99% of the NPS pollution load, with a greater contribution by lands than by livestock. The NPS pollution load was generally high in the basins of the Naeseong and Byeongseong streams, Geumho River, and downstream of the Nakdong River. For the residential/industrial NPS pollution load, the basins of the Nam and Yeong rivers were also found to be high. The water quality tended to be high in the mid- and downstream basins of the Nakdong River including the Geumho and Nam rivers. For TP, the basins of the Byeongseong and Gam streams also showed high tendencies. The WQI indicated a trend similar to the water quality. On the other hand, the excess percentage of water quality standards was generally high in the upstream basins. Within the Geumho River and Changnyeong Hapcheon Weir subbasins with the lowest standards, only the Jincheon Stream basin did not meet the water quality standards.

The Nakdong River watershed was comprised of mountainous areas (67.2%), agricultural areas (22.7%), urban areas (4.5%), and other landuses (grasslands, wetlands, bare lands, and water bodies; 5.6%). Greenhouses accounted for 2.3% of the agricultural area and the impervious area susceptible to NPS pollution was 5.0% of the total area. The impervious area was generally high in the mid- and downstream basins of the Nakdong River including the Geumho and Nam rivers. The steepest slope ratio was 60–100% (36.5% of the watershed), followed by the 30–60% slope (23.6% of the watershed). Basins with the 30–60% slope were mainly found in the upper- and midstream areas of the Nakdong River. More than 90% of the Nakdong River watershed had silty loam, loam, and sandy loam soil types. Clay loam soils accounted for only 1.7% of the total area and were mainly found in the basins of the Byeongseong Stream, Yeong and Geumho rivers, and downstream of the Nakdong River. The average annual rainfall indicated an approximately 100 mm increase from the past (1209 mm) to the future (1306 mm) while there was almost no difference in the average annual rainfall between RCP 2.6 (1305.6 mm) and RCP 8.5 (1306.8 mm) scenarios. The rainfall amount was high in the basins of the Naeseong Stream and downstream of the Nakdong River including the Nam River, both in the past and the future.

### Identification of critical source areas

**Fig. 3:**
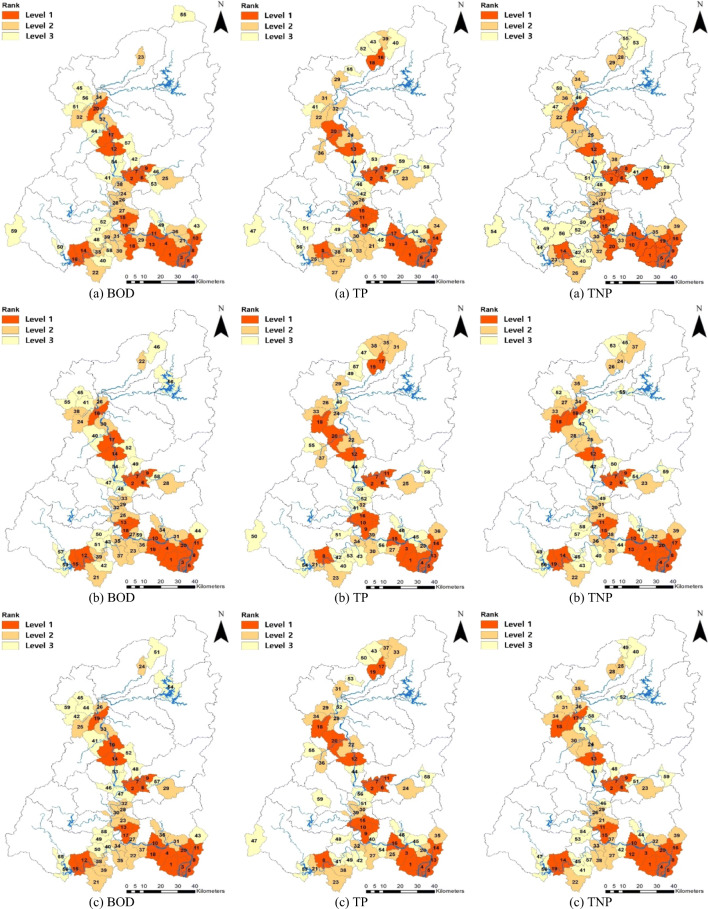
Results of the CSA identification for each variable in the present (**a**) and the future (**b** RCP2.6, **c** RCP8.5). The number and color of each basin indicate its rank and subgroup, respectively. Left, middle, and right figures from (**a**) to (**c**) mean BOD, TP, and TNP, respectively

The comprehensive analysis results of the above nine factors showed that those basins that are on or adjacent to the Nakdong River mainstream were mainly identified as CSAs in high need of NPS pollution management both in the present and the future (Fig. 3). The top 20% CSAs were mainly found in the mid- and downstream areas of the Nakdong River.

Based on the past rainfall data, the CSA results showed large agreements with respect to the spatial distributions of BOD, TP, and TNP (Fig. 3a and Table 1). The rankings of CSAs did not largely agree among BOD, TP, and TNP, but their subgroups indicated similar spatial distributions. Particularly, the basins of level 1 were the same over 80% among BOD, TP, and TNP. As the basins that are on the Nakdong River mainstream, five same basins were identified as level 1: Chilgok Weir, Before Merging of the Milyang River, Gupo Water Level Gauging Station, Nakdong River Estuary Bank, and West Nakdong River. As the basins that are adjacent to the Nakdong River mainstream, eleven tributary basins were identified as level 1: the basins of the Geumho and Nam rivers (Jincheon Stream, Downstream of the Shin Stream, Downstream, and Midstream of the Geumho River, and After Merging of the Banseong Stream) and downstream of the Nakdong River (Masuwon Water Level Gauging Station, Gyeseong Stream, Jucheon River, Hwapo Stream, Downstream of the Yangsan Stream, and Joman River). Eight of the 16 basins were revealed as important CSAs in high need of NPS pollution management, with insignificant differences even in the rankings among BOD, TN, and TNP: Joman River, Jincheon Stream, Hwapo Stream, Nakdong River Estuary Bank, West Nakdong River, Downstream of the Shin Stream, and Downstream and Midstream of the Geumho River. Many basins of the Geumho and Nam rivers were determined to be CSAs, indicating the significance of NPS pollution management. The basins of the Naeseong and Gam streams were identified to be CSAs in TP but barely in BOD.

**Table 1:** Rankings of CSAs (basins) for each variable in the present

Rank	Subgroup	BOD	TP	TNP
1	Level 1	Joman River^a^	Joman River^a^	Joman River^a^
2		Jincheon Stream^a^	Jincheon Stream^a^	Jincheon Stream^a^
3		West Nakdong River^a^	Hwapo Stream^a^	Hwapo Stream^a^
4		Hwapo Stream^a^	Nakdong River Estuary Bank^a^	Nakdong River Estuary Bank^a^
5		Nakdong River Estuary Bank^a^	Downstream of the Shin Stream^a^	West Nakdong River^a^
6		Downstream of the Shin Stream^a^	West Nakdong River^a^	Downstream of the Shin Stream^a^
7		Downstream of the Geumho River	Downstream of the Geumho River	Downstream of the Geumho River
8		Gupo Water Level Gauging Station^a^	After Merging of the Banseong Stream	Midstream of the Geumho River
9		Midstream of the Geumho River	Midstream of the Geumho River	Gupo Water Level Gauging Station^a^
10		Downstream of the Yangsan Stream^a^	Gyeseong Stream^a^	Jucheon River
11		Before Merging of the Milyang River	Masuwon Water Level Gauging Station^a^	Before Merging of the Milyang River
12		Chilgok Weir^a^	Gupo Water Level Gauging Station^a^	Chilgok Weir^a^
13		Jucheon River	Chilgok Weir^a^	Masuwon Water Level Gauging Station^a^
14		After Merging of the Banseong Stream	Downstream of the Yangsan Stream^a^	After Merging of the Banseong Stream
15		Masuwon Water Level Gauging Station^a^	Jeokpogyo Water Level Gauging Station	Gyeseong Stream^a^
16		Before Merging of the Yeongcheon River	Downstream of the Seo Stream^a^	Downstream of the Yangsan Stream^a^
17		After Merging of the Han Stream	Before Merging of the Milyang River	Omok Stream^a^
18		Gwangryeo Stream^a^	Okgye Stream^a^	Before Merging of the Wi Stream
19		Gyeseong Stream^a^	Jucheon River	Wolchon Water Level Gauging Station
20		Before Merging of the Wi Stream	Downstream of the Gam Stream^a^	Gwangryeo Stream^a^
21	Level 2	Wolchon Water Level Gauging Station	Gwangryeo Stream^a^	Jeokpogyo Water Level Gauging Station
22		Yeongcheon River	Upstream of the Byeongseong Stream^a^	Upstream of the Byeongseong Stream^a^
23		Downstream of the Seo Stream^a^	Omok Stream^a^	Before Merging of the Yeongcheon River
24		Hyeonpung Water Level Gauging Station	After Merging of the Han Stream	Changnyeong Hapcheon Weir^a^
25		Omok Stream^a^	Before Merging of the Yeongcheon River	After Merging of the Han Stream
26		Cha Stream	Cha Stream	Yeongcheon River
27		Jeokpogyo Water Level Gauging Station	Yeongcheon River	Cha Stream
28		Changnyeong Hapcheon Weir^a^	Wolchon Water Level Gauging Station	Downstream of the Seo Stream^a^
29		After Merging of the Shin Stream	Geum Stream^a^	Okgye Stream^a^
30		Haman Stream	Downstream of the Nam River	Downstream of the Nam River
31		Downstream of the Nam River	Downstream of the Byeongseong Stream^a^	Downstream of the Gam Stream^a^
32		Upstream of the Byeongseong Stream^a^	Before Merging of the Wi Stream	Haman Stream
33		Haman Changnyeong Weir	Haman Stream	After Merging of the Shin Stream
34		Sangju Weir	Upstream of the Yangsan Stream^a^	Geum Stream^a^
35		Jeongam Water Level Gauging Station	Changnyeong Hapcheon Weir^a^	Before Merging of the Wondong Stream
36		Before Merging of the Wondong Stream	Gimcheon Water Level Gauging Station	Downstream of the Byeongseong Stream^a^
37		Gumi Weir	Banseong Stream	Hyeonpung Water Level Gauging Station
38		Goryeonggyo Water Level Gauging Station	Jeongam Water Level Gauging Station	Palgeo Stream
39		Before Merging of the Haman Stream	Nakhwaam Stream	Upstream of the Yangsan Stream^a^
40	Level 3	Banseong Stream	Upstream of the Naeseong Stream^a^	Banseong Stream
41		Gangjeong Goryeong Weir	Buk Stream	Dongchon Water Level Gauging Station^a^
42		Palgeo Stream	Hyeonpung Water Level Gauging Station	Jeongam Water Level Gauging Station
43		Upstream of the Yangsan Stream^a^	Jukgye Stream	Seongju Water Level Gauging Station
44		Downstream of the Gam Stream^a^	Seongju Water Level Gauging Station	Upstream of the Nam River Dam
45		Downstream of the Ian Stream	After Merging of the Shin Stream	Haman Changnyeong Weir
46		Dongchon Water Level Gauging Station^a^	Goryeonggyo Water Level Gauging Station	Sangju Weir
47		Yugok Stream	Ram Stream	Buk Stream
48		Uiryeong Stream	Haman Changnyeong Weir	Goryeonggyo Water Level Gauging Station
49		Milyang River^a^	Yugok Stream	Shindeung Stream
50		Upstream of the Nam River Dam	Seokgyo Stream	Shinban Stream
51		Buk Stream	Shindeung Stream	Gangjeong Goryeong Weir
52		Shinban Stream	Upstream of the Seo Stream	Yugok Stream
53		Nam Stream	Palgeo Stream	Upstream of the Naeseong Stream^a^
54		Seongju Water Level Gauging Station	Before Merging of the Wondong Stream	Ram Stream
55		Hwangji Stream	Yecheon Water Level Gauging Station	Nakhwaam Stream
56		Downstream of the Byeongseong Stream^a^	Upstream of the Nam River Dam	Yang Stream
57		Han Stream	Dongchon Water Level Gauging Station^a^	Seokgyo Stream
58		Seokgyo Stream	Beginning of the Geumho River	Downstream of the Ian Stream
59		Ram Stream	Cheongtong Stream^a^	Beginning of the Geumho River

^a^Those are basins of the Nakdong River watershed reported by the MOE and KECO

The results of the CSA identification based on the future rainfall data (both RCP 2.6 and RCP 8.5) showed similar spatial patterns to those based on the past rainfall data (Fig. 3b, c and Appendix Table 2). The CSAs based on the present and the future thus showed >90% agreement with respect to the spatial distributions of all variables. In particular, the CSAs of level 1 rarely differed between the present and the future. The CSAs of the two future scenarios indicated 98% agreement with respect to all variables. The spatial distributions of the subgroups of the two future scenarios showed >85% agreement and 100% in level 1. The CSA rankings of level 1 of the future scenarios even showed >80% agreement.

### Load duration curve analysis results

**Fig. 4:**
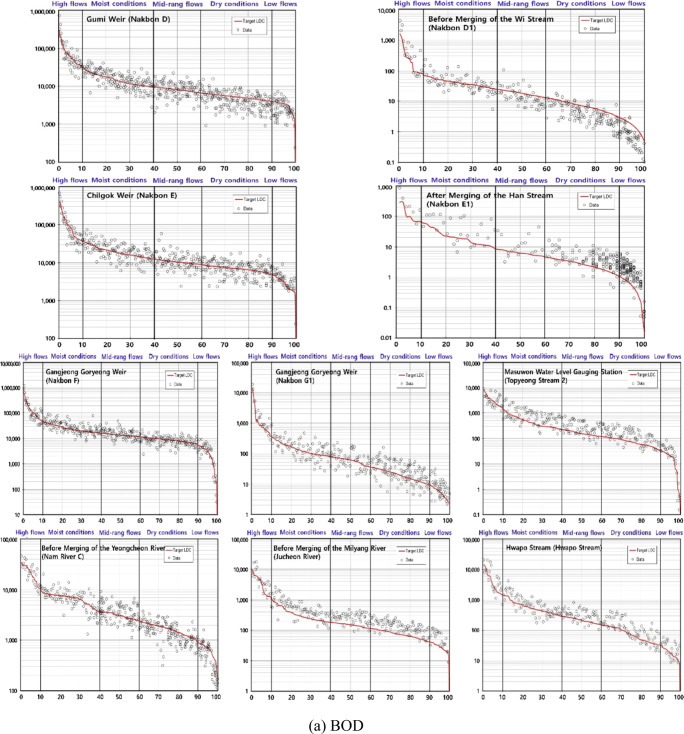

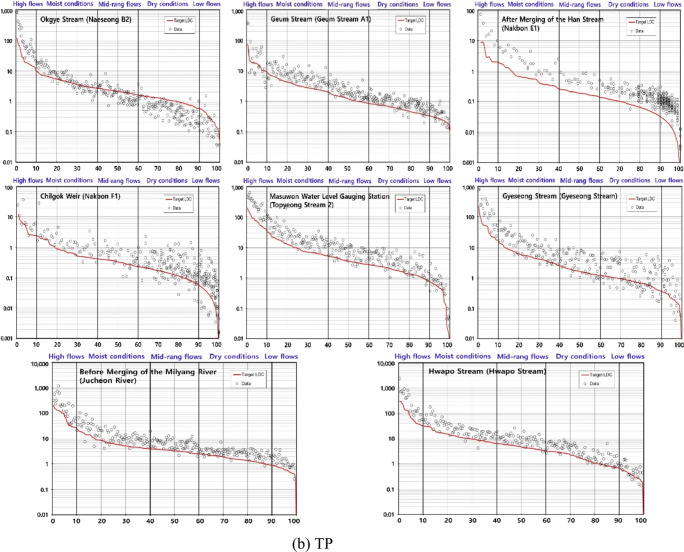
LDC results for the top 10% basins in agreement with the present CSAs. **a** BOD and **b** TP. The brackets in the figures represent the names of the Korean TMDL monitoring stations of each basin. The *x*- and *y*-axes represent the flow duration interval (%) and load (kg/day), respectively

Based on the LDC analysis, the target water quality was not met in the high flow and moist condition intervals at many Korean TMDL monitoring stations. Regarding BOD and TP, 27 and 14 and 75 and 32 monitoring stations did not meet the target water quality in the high flow and moist condition intervals, respectively. More monitoring stations were found in TP, closely related to NPS pollution. The basins including those monitoring stations fairly agreed with the present CSA results. Regarding BOD and TP, 43% and 67% and 43% and 54% of the basins corresponded to the identified CSAs in the high flow and moist condition intervals, respectively. The top 10% basins were mostly included in the present CSA results, especially level 1 (Table 2). They showed the results that did not largely satisfy the target water quality in the high flow and moist condition intervals (Fig. 4). Regarding BOD, nine of the top 10% basins were in agreement with the identified CSAs and seven basins, excluding the Gumi Weir and Gangjeong Goryeong Weir, were in level 1, indicating the high need of NPS pollution management. Regarding TP, eight of the top 10% basins corresponded with the identified CSAs and six basins were in level 1, except for the Geum Stream and After Merging of the Han Stream. In particular, four basins (Chilgok Weir, Masuwon Water Level Gauging Station, Before Merging of the Milyang River, and Hwapo Stream) were in level 1 regarding both BOD and TP, indicating areas in high need of NPS pollution management.

**Table 3:** Top 10% basins in agreement with the present CSA results based on the LDC analysis

Variable	Basin name
BOD	Before Merging of the Wi Stream^a^, Gumi Weir, After Merging of the Han Stream^a^, Chilgok Weir^a^, Gangjeong Goryeong Weir, Masuwon Water Level Gauging Station^a^, Before Merging of the Yeongcheon River^a^, Before Merging of the Milyang River^a^, Hwapo Stream^a^
TP	Okgye Stream^a^, Geum Stream, After Merging of the Han Stream, Chilgok Weir^a^, Masuwon Water Level Gauging Station^a^, Gyeseong Stream^a^, Before Merging of the Milyang River^a^, Hwapo Stream^a^

^a^The basins belong to the level 1 subgroup of the present CSAs

### Analysis of identified critical source area results

The present CSAs identified in this study showed the reliability of the results as including many basins of the Nakdong River watershed reported by the MOE and KECO. That is, the identified CSAs included 21 and 12 basins in the top 30% and level 1 for BOD, respectively, as well as 24 and 14 and 23 and 13 basins for TP and TNP, respectively (Table 1). In level 1, the basins accounted for more than 60%. In addition, the LDC analysis results showed that the basins not meeting the target water quality fairly corresponded to the present CSAs and the top 10% basins were mostly included in level 1. These results demonstrated that the identified CSAs represented the areas vulnerable to NPS pollution in the Nakdong River watershed and CSAs of level 1 should be prioritized in NPS pollution management.

The Nakdong River watershed includes urban and rural areas along the river and mainly major cities and large agricultural areas and tributaries in the mid- and downstream areas. The watershed characteristics can explain why the basins that are on or adjacent to the Nakdong River mainstream were identified as CSAs and highly ranked basins were concentrated in the mid- and especially downstream areas. The CSAs of level 1 mentioned in the “Identification of critical source areas” section are all located in the downstream areas, except for the Chilgok Weir basin and the basins of the Geumho River. They are generally areas with high imperviousness due to urban and greenhouse areas, are significantly affected by rainfall, and have clay loam soils, leading to high surface runoff. In addition, the downstream areas are prone to pollutant accumulation due to low flow velocity and gentle slope. These characteristics seemed to result in the high-ranking CSAs in the downstream areas. On the other hand, in the case of the Chilgok Weir basin toward the upstream areas, the main reasons seemed to be a high ratio of urban areas and inflow of highly polluted tributaries due to nearby large industrial complexes and livestock and agricultural activities. In particular, the high ranking of the basin was possibly due to the artificial control of flow by the Chilgok weir. Seo et al. (2019b) who examined changes in coliform bacteria at the weir stations of the Nakdong River reported a drastic increase in the coliform bacteria at the Chilgok weir station for the same reason.

The Nam and Geumho rivers are tributaries of the Nakdong River whose lengths and drainage areas are first and second, respectively, in the Nakdong River watershed. Water quality in these tributaries are generally poor, compared to other tributaries (Table 3). The Nam River subbasin has widely distributed greenhouses along the river and a high ratio of urban and agricultural areas in the upstream areas. Kim et al. (2014) and Yu et al. (2012) reported high levels of pollution in the Nam River due to NPS pollutants from livestock farms and urban and industrial areas. A number of CSAs in this subbasin was possibly because of livestock manure and fertilizer, untreated and unknown water from residential and industrial areas, and high proportions of impervious areas. The Geumho River subbasin also had many CSAs probably due to areas with high ratios of urban such as Daegu, one of the major metropolitan cities in South Korea. Kim et al. (2012) and Niraula et al. (2013) reported a high potential impact of urban areas on NPS pollution. The Omok Stream basin was the only agricultural CSA identified in the Geumho River subbasin, which seemed due to agricultural activities in an exceptionally high ratio of agricultural areas (ranked 2nd among all 195 basins). The Geumho River subbasin had a higher ratio of clay loam soils than the other subbasins (Fig. 2h), which could be one of the reasons for many CSAs in this subbasin. Jung et al. (2016) and Seo et al. (2019b) reported that dense industrial complexes and wastewater treatment plants and agricultural areas in this subbasin might have negative impacts on water quality of the Geumho River.

**Table 4:** Mean and standard deviations for the water quality of the major tributaries that flow into the Nakdong River. The rainy and dry seasons are the months from June to September and remaining months, respectively

Tributary	BOD			COD			TP			SS		
	Total	Rainy season	Dry season	Total	Rainy season	Dry season	Total	Rainy season	Dry season	Total	Rainy season	Dry season
Banbyeon Stream	1.2 ± 0.46	1.3 ± 0.52	1.1 ± 0.39	5.5 ± 1.39	6.2 ± 1.89	5.1 ± 0.85	0.023 ± 0.02	0.033 ± 0.02	0.017 ± 0.01	6.6 ± 10.80	11.1 ± 17.60	4.3 ± 2.64
Naeseong Stream	0.9 ± 0.50	1.0 ± 0.42	0.8 ± 0.52	3.5 ± 1.68	4.4 ± 2.18	3.0 ± 1.13	0.070 ± 0.05	0.096 ± 0.06	0.056 ± 0.03	17.8 ± 28.67	30.4 ± 43.09	11.6 ± 14.15
Yeong River	1.4 ± 0.66	1.4 ± 0.66	1.4 ± 0.66	3.9 ± 1.01	4.3 ± 1.09	3.7 ± 0.91	0.036 ± 0.03	0.046 ± 0.03	0.031 ± 0.02	7.7 ± 12.81	14.1 ± 19.87	4.5 ± 4.55
Byeongseong Stream	1.6 ± 0.78	1.7 ± 0.73	1.6 ± 0.81	5.1 ± 1.46	5.9 ± 1.51	4.8 ± 1.29	0.110 ± 0.08	0.124 ± 0.07	0.102 ± 0.08	14.4 ± 19.53	21.4 ± 22.05	10.9 ± 17.23
Wi Stream	1.9 ± 0.82	2.2 ± 0.71	1.7 ± 0.82	6.0 ± 1.89	7.7 ± 1.39	5.2 ± 1.53	0.043 ± 0.03	0.067 ± 0.03	0.032 ± 0.02	12.7 ± 14.53	19.6 ± 16.63	9.2 ± 12.05
Gam Stream	1.3 ± 0.57	1.3 ± 0.56	1.3 ± 0.57	4.6 ± 1.31	5.3 ± 1.70	4.3 ± 0.90	0.105 ± 0.07	0.113 ± 0.06	0.101 ± 0.08	13.2 ± 13.43	18.6 ± 18.47	10.5 ± 8.98
Geumho River	3.6 ± 1.34	4.0 ± 1.18	3.3 ± 1.36	8.7 ± 1.62	9.3 ± 1.61	8.5 ± 1.57	0.275 ± 0.24	0.251 ± 0.17	0.287 ± 0.27	13.6 ± 11.17	19.9 ± 14.16	10.5 ± 7.64
Hoe Stream	1.3 ± 0.56	1.5 ± 0.58	1.2 ± 0.53	4.1 ± 1.27	5.0 ± 1.43	3.7 ± 0.88	0.040 ± 0.03	0.063 ± 0.04	0.028 ± 0.01	8.7 ± 10.45	13.0 ± 13.72	6.5 ± 7.56
Hwang River	0.7 ± 0.30	0.9 ± 0.25	0.7 ± 0.30	3.5 ± 0.76	3.9 ± 0.82	3.3 ± 0.62	0.033 ± 0.02	0.046 ± 0.02	0.027 ± 0.01	15.3 ± 11.21	19.8 ± 11.79	13.1 ± 10.26
Nam River	2.7 ± 1.13	3.1 ± 1.02	2.5 ± 1.14	6.1 ± 1.68	7.0 ± 1.12	5.7 ± 1.75	0.074 ± 0.04	0.090 ± 0.03	0.067 ± 0.05	15.9 ± 10.58	23.4 ± 12.80	12.2 ± 6.71
Milyang River	2.2 ± 1.10	2.5 ± 1.01	2.1 ± 1.14	4.9 ± 1.44	5.6 ± 1.41	4.6 ± 1.33	0.072 ± 0.05	0.071 ± 0.04	0.072 ± 0.06	9.6 ± 7.49	13.1 ± 11.07	7.9 ± 3.86
Yangsan Stream	4.0 ± 1.96	3.6 ± 1.43	4.2 ± 2.16	7.6 ± 2.03	7.4 ± 2.06	7.6 ± 2.02	0.194 ± 0.18	0.140 ± 0.09	0.221 ± 0.21	18.0 ± 34.63	19.8 ± 12.98	17.1 ± 41.47

However, rainfall is, above all else, an influential factor inducing NPS pollution (Huang et al. 2015), which we found in good agreement in this study. The water quality of major tributaries of the Nakdong River presented the impact of rainfall, generally indicating higher concentrations and wider variations during rainy seasons than dry seasons (Table 3). The relative significance of individual factors that are considered in identifying CSAs was evaluated by comparing CSA result considering the full nine factors with nine subsets of CSA results considering only eight factors. Among those subsets, the CSA result considering eight factors but the rainfall factor was excluded presented the largest difference (Table 4). In addition, as large amounts of rainfall occurred in the mid- and downstream areas of the Nakdong River as well as in the Naeseong Stream subbasin (Fig. 2i), the high-ranking CSAs showing up in the mid- and downstream areas indicated a good agreement between the distribution of CSAs and the spatial patterns of rainfall. Regarding TP, several basins of the Naeseong Stream subbasin were identified as CSAs, similar to the rainfall distribution. The impact of rainfall in the identification of CSAs also provided a clue on future CSAs such as the Byeongseong Stream, Andong Dam, and Downstream of the Andong Dam subbasins. These subbasins will be in greater needs for NPS management given that they are expected to get 15-18% increases in rainfall in the future.

**Table 5:** Impact of each factor on the identification of CSAs (in %). The value represents the ratio of the same basins identified as CSAs before and after the elimination of each factor and the range of the value refers to the results based on BOD, TP, and TNP

Rank	−Factor 1^a^	−Factor 2	−Factor 3	−Factor 4	−Factor 5	−Factor 6	−Factor 7	−Factor 8	−Factor 9
Total (top 30%)	89.8–94.9	98.3–100.0	88.1–93.2	89.8–91.5	84.7–88.1	89.8–93.2	88.1	94.9–96.6	74.6–84.7
Level 1	90.0–95.0	95.0–100.0	90.0–95.0	85.0–90.0	90.0–95.0	85.0–90.0	90.0–95.0	90.0–95.0	80.0–85.0
Level 2	68.4–84.2	89.5–100.0	68.4–84.2	57.9–73.7	73.7–78.9	68.4–73.7	57.9–78.9	84.2	26.3–36.8
Level 3	55.0–65.0	95.0	55.0–65.0	50.0–60.0	45.0–60.0	55.0–60.0	35.0–55.0	75.0–90.0	10.0–20.0

^a^“−” indicates the elimination of the factor, with (1) NPS pollution load per unit area, (2) residential/industrial NPS pollution load per unit area, (3) mean water quality, (4) excess percentage of water quality standards, (5) water quality index, (6) impervious area, (7) soil slope, (8) topsoil type, and (9) rainfall

Doppler et al. (2014) reported that the CSAs of NPS pollution could vary depending on rainfall. The rainfall factor largely affected the identification of CSAs as described above. However, it did not cause large discrepancies in the spatial distribution of CSAs among the present and two future (RCP 2.6 and RCP 8.5) scenarios. It seemed because there was little difference in rainfall among those scenarios. The future scenarios do not provide detailed rainfall information, such as duration and intensity; thus, the amount of rainfall was only used in this study to identify CSAs. More research should be carried out on statistically relating CSA identification and various future climatic properties to properly identify future NPS pollution. The results of this study indicated that the present CSAs of NPS pollution will likely be the CSAs of NPS pollution in the future. This means that (1) a focus should be placed on the present CSAs in preparation for future NPS pollution; (2) extra efforts should be made to manage NPS pollution in highly ranked CSAs (level 1) in both the present and the future; and (3) the CSAs identified only in the future scenarios should not be overlooked to prevent uncontrolled NPS pollution in these areas in the future.

## Conclusions

Identification of CSAs is the first step toward an effective management of NPS pollution. In this study, CSAs that are in need of NPS pollution management in the present and the future were identified in the Nakdong River watershed, South Korea. The identified CSAs were mainly distributed along the Nakdong River mainstream. Areas in the mid- and downstream of the Nakdong River were identified as CSAs in need of substantial management in both the present and the future. Particularly, differences in the distribution of CSAs between the present and the future were found insignificant. The results demonstrated that future management of NPS pollution should be planned out based on the present distribution of CSAs regardless of the impact of climate change in the Nakdong River watershed. However, it is obviously important to consider the impact of climate change in predicting and preparing for future NPS pollution. A variety of research related to climate change is needed to allow an effective management of NPS pollution in the future. It is also necessary to establish step-by-step local strategies of NPS pollution management under climate change, such as investigating major pollutants, setting up goals, and suggesting measures for identified CSAs under changing climate conditions. Such studies will provide regional policy makers with information on NPS pollution management, facilitating the proper management of regions in the present and the future.

## Appendix

**Table 2:** Rankings of CSAs (basins) for each variable in the future based on (a) RCP 2.6 and (b) RCP 8.5

Rank	Subgroup	(a) Based on future rainfall data (RCP 2.6)		
		BOD	TP	TNP
1	Level 1	Joman River	Joman River	Joman River
2		Jincheon Stream	Jincheon Stream	Jincheon Stream
3		West Nakdong River	Hwapo Stream	Hwapo Stream
4		Hwapo Stream	West Nakdong River	West Nakdong River
5		Nakdong River Estuary Bank	Nakdong River Estuary Bank	Nakdong River Estuary Bank
6		Downstream of the Shin Stream	Downstream of the Shin Stream	Downstream of the Shin Stream
7		Downstream of the Geumho River	Downstream of the Geumho River	Downstream of the Geumho River
8		Gupo Water Level Gauging Station	After Merging of the Banseong Stream	Gupo Water Level Gauging Station
9		Midstream of the Geumho River	Gyeseong Stream	Midstream of the Geumho River
10		Before Merging of the Milyang River	Masuwon Water Level Gauging Station	Before Merging of the Milyang River
11		Downstream of the Yangsan Stream	Midstream of the Geumho River	Masuwon Water Level Gauging Station
12		After Merging of the Banseong Stream	Chilgok Weir	Chilgok Weir
13		Masuwon Water Level Gauging Station	Gupo Water Level Gauging Station	Jucheon River
14		Chilgok Weir	Downstream of the Yangsan Stream	After Merging of the Banseong Stream
15		Before Merging of the Yeongcheon River	Before Merging of the Milyang River	Gyeseong Stream
16		Before Merging of the Wi Stream	Jeokpogyo Water Level Gauging Station	Before Merging of the Wi Stream
17		After Merging of the Han Stream	Downstream of the Seo Stream	Downstream of the Yangsan Stream
18		Gyeseong Stream	Upstream of the Byeongseong Stream	Upstream of the Byeongseong Stream
19		Jucheon River	Okgye Stream	Before Merging of the Yeongcheon River
20		Wolchon Water Level Gauging Station	Downstream of the Gam Stream	Wolchon Water Level Gauging Station
21	Level 2	Yeongcheon River	Before Merging of the Yeongcheon River	Jeokpogyo Water Level Gauging Station
22		Downstream of the Seo Stream	After Merging of the Han Stream	Yeongcheon River
23		Gwangryeo Stream	Yeongcheon River	Omok Stream
24		Upstream of the Byeongseong Stream	Before Merging of the Wi Stream	Downstream of the Seo Stream
25		Jeokpogyo Water Level Gauging Station	Omok Stream	After Merging of the Han Stream
26		Sangju Weir	Downstream of the Byeongseong Stream	Okgye Stream
27		Haman Changnyeong Weir	Jucheon River	Downstream of the Byeongseong Stream
28		Omok Stream	Wolchon Water Level Gauging Station	Downstream of the Gam Stream
29		Cha Stream	Geum Stream	Changnyeong Hapcheon Weir
30		Gumi Weir	Gwangryeo Stream	Gwangryeo Stream
31		Before Merging of the Wondong Stream	Upstream of the Naeseong Stream	Cha Stream
32		Changnyeong Hapcheon Weir	Cha Stream	Before Merging of the Wondong Stream
33		Hyeonpung Water Level Gauging Station	Buk Stream	Buk Stream
34		Milyang River	Downstream of the Nam River	Sangju Weir
35		Downstream of the Nam River	Nakhwaam Stream	Geum Stream
36		After Merging of the Shin Stream	Upstream of the Yangsan Stream	Downstream of the Nam River
37		Haman Stream	Gimcheon Water Level Gauging Station	Upstream of the Naeseong Stream
38		Buk Stream	Jukgye Stream	Haman Changnyeong Weir
39		Jeongam Water Level Gauging Station	Haman Changnyeong Weir	Upstream of the Yangsan Stream
40	Level 3	Downstream of the Gam Stream	Banseong Stream	Haman Stream
41		Downstream of the Byeongseong Stream	Changnyeong Hapcheon Weir	Milyang River
42		Banseong Stream	Jeongam Water Level Gauging Station	Seongju Water Level Gauging Station
43		Before Merging of the Haman Stream	Haman Stream	Banseong Stream
44		Upstream of the Yangsan Stream	Seongju Water Level Gauging Station	After Merging of the Shin Stream
45		Downstream of the Ian Stream	Before Merging of the Wondong Stream	Nakhwaam Stream
46		Upstream of the Naeseong Stream	Milyang River	Jeongam Water Level Gauging Station
47		Gangjeong Goryeong Weir	Upstream of the Seo Stream	Gumi Weir
48		Goryeonggyo Water Level Gauging Station	Sangju Weir	Upstream of the Nam River Dam
49		Palgeo Stream	Yecheon Water Level Gauging Station	Hyeonpung Water Level Gauging Station
50		Yugok Stream	Ram Stream	Palgeo Stream
51		Uiryeong Stream	Yugok Stream	Wi Stream
52		Han Stream	Hyeonpung Water Level Gauging Station	Downstream of the Ian Stream
53		Nam River Dam	Seokgyo Stream	Jukgye Stream
54		Seongju Water Level Gauging Station	Nam River Dam	Dongchon Water Level Gauging Station
55		Upstream of the Ian Stream	Jikjisa Stream	Downstream of the Andong Dam Balancing Reservoir
56		Andong Dam	After Merging of the Shin Stream	Nam River Dam
57		Upstream of the Nam River Dam	Upstream of the Han Stream	Yugok Stream
58		Dongchon Water Level Gauging Station	Beginning of the Geumho River	Shinban Stream
59		Imhaejin Water Level Gauging Station	Goryeonggyo Water Level Gauging Station	Beginning of the Geumho River
Rank	Subgroup	(b) Based on future rainfall data (RCP 8.5)		
		BOD	TP	TNP
1	Level 1	Joman River	Joman River	Joman River
2		Jincheon Stream	Jincheon Stream	Jincheon Stream
3		West Nakdong River	Hwapo Stream	Hwapo Stream
4		Hwapo Stream	West Nakdong River	West Nakdong River
5		Nakdong River Estuary Bank	Nakdong River Estuary Bank	Nakdong River Estuary Bank
6		Downstream of the Shin Stream	Downstream of the Shin Stream	Downstream of the Shin Stream
7		Downstream of the Geumho River	Downstream of the Geumho River	Downstream of the Geumho River
8		Gupo Water Level Gauging Station	After Merging of the Banseong Stream	Gupo Water Level Gauging Station
9		Midstream of the Geumho River	Gyeseong Stream	Midstream of the Geumho River
10		Before Merging of the Milyang River	Masuwon Water Level Gauging Station	Before Merging of the Milyang River
11		Downstream of the Yangsan Stream	Midstream of the Geumho River	Masuwon Water Level Gauging Station
12		After Merging of the Banseong Stream	Chilgok Weir	Jucheon River
13		Masuwon Water Level Gauging Station	Gupo Water Level Gauging Station	Chilgok Weir
14		Chilgok Weir	Downstream of the Yangsan Stream	After Merging of the Banseong Stream
15		Before Merging of the Yeongcheon River	Jeokpogyo Water Level Gauging Station	Gyeseong Stream
16		After Merging of the Han Stream	Before Merging of the Milyang River	Downstream of the Yangsan Stream
17		Gyeseong Stream	Downstream of the Seo Stream	Before Merging of the Wi Stream
18		Jucheon River	Upstream of the Byeongseong Stream	Upstream of the Byeongseong Stream
19		Before Merging of the Wi Stream	Okgye Stream	Before Merging of the Yeongcheon River
20		Wolchon Water Level Gauging Station	Downstream of the Gam Stream	Wolchon Water Level Gauging Station
21	Level 2	Yeongcheon River	Before Merging of the Yeongcheon River	Jeokpogyo Water Level Gauging Station
22		Gwangryeo Stream	After Merging of the Han Stream	Yeongcheon River
23		Jeokpogyo Water Level Gauging Station	Yeongcheon River	Omok Stream
24		Downstream of the Seo Stream	Omok Stream	After Merging of the Han Stream
25		Upstream of the Byeongseong Stream	Jucheon River	Downstream of the Seo Stream
26		Sangju Weir	Wolchon Water Level Gauging Station	Changnyeong Hapcheon Weir
27		Haman Changnyeong Weir	Gwangryeo Stream	Gwangryeo Stream
28		Cha Stream	Before Merging of the Wi Stream	Okgye Stream
29		Omok Stream	Downstream of the Byeongseong Stream	Cha Stream
30		Changnyeong Hapcheon Weir	Cha Stream	Downstream of the Gam Stream
31		Before Merging of the Wondong Stream	Geum Stream	Downstream of the Byeongseong Stream
32		Hyeonpung Water Level Gauging Station	Downstream of the Nam River	Before Merging of the Wondong Stream
33		Gumi Weir	Upstream of the Naeseong Stream	Downstream of the Nam River
34		Downstream of the Nam River	Buk Stream	Buk Stream
35		Haman Stream	Upstream of the Yangsan Stream	Geum Stream
36		Milyang River	Gimcheon Water Level Gauging Station	Sangju Weir
37		After Merging of the Shin Stream	Nakhwaam Stream	Haman Changnyeong Weir
38		Jeongam Water Level Gauging Station	Banseong Stream	Haman Stream
39		Banseong Stream	Changnyeong Hapcheon Weir	Upstream of the Yangsan Stream
40	Level 3	Before Merging of the Haman Stream	Haman Changnyeong Weir	Upstream of the Naeseong Stream
41		Downstream of the Gam Stream	Jeongam Water Level Gauging Station	Banseong Stream
42		Buk Stream	Haman Stream	After Merging of the Shin Stream
43		Upstream of the Yangsan Stream	Jukgye Stream	Seongju Water Level Gauging Station
44		Downstream of the Byeongseong Stream	Seongju Water Level Gauging Station	Milyang River
45		Downstream of the Ian Stream	Before Merging of the Wondong Stream	Jeongam Water Level Gauging Station
46		Gangjeong Goryeong Weir	Milyang River	Hyeonpung Water Level Gauging Station
47		Goryeonggyo Water Level Gauging Station	Ram Stream	Upstream of the Nam River Dam
48		Palgeo Stream	Yugok Stream	Palgeo Stream
49		Yugok Stream	Seokgyo Stream	Nakhwaam Stream
50		Uiryeong Stream	Upstream of the Seo Stream	Gumi Weir
51		Upstream of the Naeseong Stream	Hyeonpung Water Level Gauging Station	Dongchon Water Level Gauging Station
52		Han Stream	Sangju Weir	Downstream of the Andong Dam Balancing Reservoir
53		Seongju Water Level Gauging Station	Yecheon Water Level Gauging Station	Yugok Stream
54		Andong Dam	After Merging of the Shin Stream	Shinban Stream
55		Upstream of the Nam River Dam	Jikjisa Stream	Downstream of the Ian Stream
56		Nam River Dam	Goryeonggyo Water Level Gauging Station	Nam River Dam
57		Dongchon Water Level Gauging Station	Nam River Dam	Seokgyo Stream
58		Shinban Stream	Beginning of the Geumho River	Wi Stream
59		Upstream of the Ian Stream	Gaya Stream	Beginning of the Geumho River
